# Reasons patients leave their nearest healthcare service to attend Karen Park Clinic, Pretoria North

**DOI:** 10.4102/phcfm.v5i1.559

**Published:** 2013-10-25

**Authors:** Agnes T. Masango-Makgobela, Indiran Govender, John V. Ndimande

**Affiliations:** 1 Department of Family Medicine and Primary Health Care, Medunsa Campus of the University of Limpopo, South Africa

## Abstract

**Background:**

Many patients move from one healthcare provider or facility to another, disturbing the continuity that enhances holistic patient care.

**Objectives:**

To investigate the reasons given by patients for attending Karen Park Clinic rather than the clinic nearest to their homes.

**Methods:**

A cross-sectional descriptive study was conducted during 2010. Three hundred and fifty patients attending Karen Park Clinic were given questionnaires to complete, with the following variables: place of residence; previous attendance at the clinic nearest their home; services available at their nearest clinic; and their willingness to attend their nearest clinic in future.

**Results:**

Respondents were from Soshanguve (153; 43.7%), Mabopane (92; 26.3%), Garankuwa (29; 8.3%) and Hebron (20; 5.7%) and most were women (271; 77.4%) aged 26–45 (177; 50.6%). Eighty per cent (281) of the patients had visited their nearest clinic previously and 54 of these (19.2%) said they would not return. The reasons for this were: long waiting time (88; 25.1%); long queues (84; 24%); rude staff (60; 17%); and no medication (39; 11.1%).

**Conclusion:**

The majority of patients who had attended their nearest clinic were adamant that they would not return. It is necessary to reduce waiting times, thus reducing long queues. This can be achieved by having adequate, satisfied healthcare providers to render a quality service and by organising training for management. Patients can thus be redirected to their nearest clinic and the health centre's capacity can be increased by procuring adequate drugs. There is a need to follow up on patients’ complaints about staff attitudes.

## Abstrait

**Les raisons pour lesquelles les patients quittent leur service de santé le plus proche pour aller à la Clinique Karen Park à Pretoria Nord**

**Contexte:**

De nombreux patients vont d'un fournisseur ou établissement de santé à l'autre, perturbant la continuité qui améliore les soins complets du patient.

**Objectifs:**

Enquêter sur les raisons invoquées par les patients et pour lesquelles ils fréquentent la Clinique Karen Park plutôt que la clinique la plus proche de chez eux.

**Méthodes:**

Une étude descriptive transversale a été menée en 2010. Trois cent cinquante patients fréquentant la Clinique Karen Park se sont vus administrer un questionnaire avec les variables suivantes: lieu de résidence; visite antérieure à la clinique la plus proche de chez eux; services disponibles à la clinique la plus proche; et leur acceptation d'aller à la clinique la plus proche à l'avenir.

**Résultats:**

Les personnes interrogées venaient de Soshanguve (153; 43,7%), Mabopane (92; 26,3%), Garankuwa (29; 8,3%) et Hebron (20; 5,7%) et la plupart étaient des femmes (271; 77,4%) âgées de 26 à 45 ans (177; 50,6%). Quatre-vingt pour cent (281) des patients s’étaient auparavant rendus dans la clinique la plus proche de chez eux et 54 d'entre eux (19,2%) affirmaient qu'ils n'y retourneraient pas. Les raisons invoquées étaient: un temps d'attente long (88; 25,1%); de longues queues (84; 24%) un personnel impoli (60; 17%); et pas de médicaments (39; 11,1%).

**Conclusion:**

La majorité des patients ayant fréquenté la clinique la plus proche étaient catégoriques sur le fait qu'ils n'y retourneraient pas. Il est nécessaire de réduire les temps d'attente et donc les longues queues. Ceci peut être réalisé en ayant des fournisseurs de soins adéquats et satisfaits fournissant un service de qualité et en organisant une formation des cadres. Les patients peuvent ainsi être redirigés vers la clinique la plus proche de chez eux et la capacité du centre de soins peut être augmentée par la fourniture de médicaments adéquats. Il existe un besoin de suivi des plaintes des patients sur l'attitude du personnel.

## Introduction

Community-based primary healthcare is the mainstay of healthcare delivery to persons in South Africa. In South Africa, primary healthcare must be accessible to the vast majority of the population in order to be successful. Poor access to primary healthcare is associated with adverse pregnancy outcomes, infant mortality, decreased vaccination coverage and decreased contraceptive use. Inaccessibility of clinics may also affect adherence to treatment regimens for chronic diseases. The successful attainment of at least three of the United Nation's Millennium Development Goals (reduce child mortality; improve maternal health; combat HIV, malaria and other diseases) is contingent upon improved access to and acceptability of primary healthcare.^1^ Patient satisfaction with primary care reflects a combination of the quality of care provided by the physician and the quality of the organisational system in which the care takes place.^2^

This makes the placement of healthcare facilities in deprived settings particularly important and it is therefore vital that facilities are sited in such a way that as many people as possible have access to the services they offer. However, deciding how to allocate primary healthcare resources is difficult and can be based on many epidemiological, sociogeographic and ethical criteria.^1^

Healthcare in South Africa is still different for Black and White South Africans, with Black South Africans having less accessibility. In total, 6.8 million children have to travel more than 30 minutes to reach their usual healthcare service provider. Nationally, physical access to health services remained relatively constant between 2002 and 2010. There is considerable variation between provinces with regard to the quality of and ease of access to healthcare. In the Western Cape, the majority (62%) of households take less than 15 minutes to reach their nearest health facility. In the Eastern Cape, this is possible for only 28% of households.^3^

The leading cause of patients’ failure to attend follow-up appointments is reported to be transport availability and cost.^4^ In South Africa, the average travel time for patients attending a clinic is two hours for a return trip and most use a minibus taxi. The median time taken to travel (return trip) to the clinic on foot or by public transport is 90 minutes, by own car 60 minutes and by other private car (a hired car or catching a lift) 120 minutes. Individuals hire these cars, or wait for them at pick-up points, or obtain lifts on their way to the destination point.^5^

Self-reported reasons for not using the closest clinic were that the clinic they attended was closer to their workplace, that they were offered good service at the present clinic (perception of quality care) and that the nearest clinic did not give correct treatment or had too many patients, long queues with long waiting times and non-availability of medicines. Other reasons for not changing clinics to the one closest to them were that they did not know how to change clinics; treatment for their condition was almost completed at the more distant clinic and they did not want to change clinics at that stage; and they had changed their place of residence and still continued with the clinic where they had always gone. Patients often change facilities due to referrals to other institutions.^6^ This was confirmed in Hlabisa, South Africa, where reasons for patients not using the clinic closest to home included better quality of care, shorter waiting time and queues, as well as closeness to place of work.^5^

Many patients in South Africa receiving TB and HIV care stated that people in the community judged them negatively with regard to their disease. They therefore made use of clinics that were located far from their homes. Patients who came for family planning did not use the nearest clinic as these patients were concerned about their confidentiality.^7^ However, these patients require continuity of care. This survey was done amongst adolescent women in the USA, but is equally relevant in the South African context.

Patients move from one clinic to another because of their dissatisfaction with the services they receive. Adequate staffing at a health centre reduces patients’ waiting time.^8^ The shortage of healthcare providers is one of the reasons for longer waiting times. Therefore, people will continue to move from one clinic to another purely to avoid long waiting times. The increased number of patients attending clinics which do not have the resources to cope results in increased waiting times and thus a vicious cycle is established.^9^

In choosing a doctor, people look at professionally-relevant factors (e.g. the appearance of the office and whether the doctor is certified with a professional Board) and management practices.^10^ Patients also leave their healthcare service when their doctor with whom they had a good relationship has moved away, died or retired, or when the client has relocated.^11^ In patients with chronic conditions, the doctor–patient relationship is important for continuity of care and improves the outcome.^12^ It is thus important for the client to be near a healthcare service so as to enable regular and easy access. Patients with chronic conditions default on their follow-up visits because of overcrowded clinics, prolonged waiting times and the probability of seeing a different doctor at every visit.^12^ Continuity of care is associated with a reduction in resource utilisation and costs.^13^ The leading predictors of patients’ loyalty to their doctor were: patient trust as a result of the doctor knowing his or her patient, good communication and good interpersonal understanding.^11^ Most patients who seek care for physical symptoms have more than one expectation and if these are not met they are dissatisfied with the physician. If their expectations are not met, patients are not satisfied^6^ and they then move to another health facility.

The World Health Organization (WHO) and the South African Department of Health continually emphasise accessible quality service delivery.^2^ However, in order to deliver on this, patients should attend the clinics nearest to their homes as resources are allocated depending on population density. This study sought to understand why people leave their nearest healthcare service in order to attend Karen Park Clinic, Pretoria North.

## Methods

### Study design

This study followed a cross-sectional descriptive design.

### Setting

Karen Park Clinic (KPC) is in Akasia, north of Pretoria. The clinic renders services to approximately 200 patients daily. The clinic is situated near a shopping complex and municipality offices. Other large adjoining townships in neighbouring subdistricts are Soshanguve, Mabopane, Garankuwa and Hebron. Many patients from these townships use KPC; indeed, most of the patients who attend KPC are not from Akasia and the majority of clients using KPC live in Soshanguve. KPC is in the subdistrict of Mesweding which is part of the Tshwane District of the Gauteng Province. According to demographics, only patients who live in the Metsweding and/or Karen Park area should be using KPC.

### Study population

The study population were all patients attending KPC whose home address was not Akasia (the area served by KPC). These patients had clinics closer to their homes that they could have used.

### Study sample

The sample included adults, over 18 years of age, who did not live in the Akasia area and who gave their consent to participate. EpiInfo (version 6.3) was used to calculate the required sample size. With a 95% confidence interval and a standard error margin of 0.01, the required sample size was calculated to be 350 patients. The questionnaires were distributed to 350 patients, using a table of random numbers. This was done by using the daily register where patients register for the day before seeing the healthcare professional. Patients meeting the inclusion criteria were selected daily from the list until the required sample was reached.

### Data collection

The study was conducted from 21 June to 29 June 2010. The instrument used to collect the data was a questionnaire in both English and Setswana. The questionnaire consisted of questions with tick boxes and was self-administered.

### Data analysis

The quantitative data were analysed using the Statistics Package for the Social Sciences (SPSS) version 17.0. Cross-tabulations were done to determine the relationship between the predictor variables and the responses and the data were then analysed using descriptive statistics.

### Ethical considerations

Informed consent was obtained from all the participants. Confidentiality and anonymity were ensured, with no identifying details of the clients being recorded. Ethical clearance was obtained from the Medunsa Research and Ethics committee (MREC/M/154/2009).

## Results

There were 334 respondents, the majority of whom (153; 43.7%) lived in Soshanguve, were female (271; 77.4%) and were aged between 26 and 45 years (177; 50.6%). One hundred and eighty-three (52.3%) respondents were employed, 110 (31.4%) of whom worked in Akasia. Table 1 contains the demographic data from this study.

**TABLE 1:** Characteristics of respondents

Variables	Number	%
**Where do you live?**		
Soshanguve	153	43.7
Mabopane	92	26.3
Garankuwa	29	8.3
Hebron	20	5.7
Other (specify)	40	15.4
**Total**	**334**	**-**
**Gender**		
Male	63	22.0
Female	271	77.4
**Total**	**334**	**-**
**Age group**		
18–25 years	101	28.9
26–45 years	177	50.6
> 46 years	56	18.6
**Total**	**334**	**-**
**Are you employed?**		
Yes	183	52.3
No	151	43.1
**Total**	**334**	**-**
**Work Area**		
Akasia	110	31.4
Pretoria centre	51	14.6
Other (specify)	71	20.3
**Total**	**232** *	**-**
**Ever visited nearest clinic?**		
Yes	281	84.1
No	53	15.9
**Total**	**334**	**-**
**How many times?**		
Once	87	24.9
Twice	93	26.6
More than twice	105	30.0
**Total**	**285** *	**-**
**Fee payable at your clinic?**		
Yes	41	11.7
No	281	80.3
**Total**	**322** *	**-**
**Method of transport to clinic**		
Walk	83	24.8
Own car	38	11.4
Public transport	213	63.8
**Total**	**334**	**-**
**Operating hours of nearest clinics**		
07h30 to 16h00	237	67.7
07h00 to 19h00	19	5.4
24 hours	57	16.3
**Total**	**313** *	**-**
**Will you return to your nearest clinic?**		
Yes	131	39.7
No	191	54.6
**Total**	**322** *	**-**
**Reason for not attending nearest clinic?**		
No medication	39	11.1
Long queues	84	24
Rude staff	60	17
Long waiting times	88	25
Other reason	63	18
**Total**	**334**	**-**
**Reason for choosing KPC?**		
Friends and/or family	243	69.4
Hospital and/or nurses	37	10.6
Others	54	16
**Total**	**334**	**-**
**Reason for visit at KPC?**		
Illness	154	44.3
Family planning	44	12.6
Chronic disease	52	15.1
Completion of forms	2	0.6
Other reason	82	23.7
**Total**	**334**	**-**

* Missing data are due to some respondents not answering all questions, thus totals are sometimes less than 334.

Most respondents (280; 80%) had visited their nearest clinic at least once and 100 (30%) had been cared for at the clinic more than twice. The majority of the respondents (281; 80.3%) said that there were no fees payable for consultation at their clinic (Table 1).

Table 1 also shows that the majority of the respondents (243; 69.4%) stated that the reason they chose KPC was that they were referred to the clinic by friends and family members, whilst only 37 (10.6%) chose KPC because of the doctors and nurses working there.

All the nearest clinics were visited only once, except in the case of the participants from the Soshanguve area who had visited their local clinic twice. The majority (213; 63.8%) of the participants used public transport to visit their nearest clinic.

Most participants (191; 54.6%) did not want to return to their nearest clinic, regardless of their area of residence. This is disturbing as this affects planning since resources to clinics are allocated based on the number of people living close to the clinic. Most clients (154; 44.3%) attended the Karen Park Clinic because they were ill rather than for reasons related to health promotion and disease preventative care.

## Discussion

The participants from Soshanguve had clinics near their homes and they had visited them more than twice. Some of these clinics were open 24 hours per day. The clinics closest to the patients were fully functional, as almost all services were available in their health centres, they did not turn patients away, they had the same medication available as KPC and they were open daily.

The majority of patients required public transport even when attending the clinic nearest to their homes. However, in order to reach KPC they had to travel much further, sometimes using two mini buses. It seems, therefore, that transport was *not* a factor in preventing people from using their nearest healthcare services, although other studies have found that transport was a *major* factor in preventing people from getting to clinics.^4, 14^

The majority of patients (243; 69.4%) who choose to seek medical attention at KPC do so on recommendation from their friends and family members. This suggests that patients using KPC are satisfied with the services provided and feel comfortable with referring their friends and family members to this clinic. This suggests that the quality of services offered by a clinic attracts patients from outside its catchment area.

It was significant that the majority would not go back to their nearest clinic (*p* = 0.004). The reasons given were the long waiting periods and the large number of patients compared with availability of healthcare staff. Long waiting times were a common problem^15^ and most of the clinics close at 4 pm, causing further problems.

Another reason for bypassing their closest clinic was rude staff. Bad staff attitudes may be a result of a number of reasons including fatigue, dissatisfaction with their job or being unhappy with their salaries. Health providers are more satisfied if there is adequate equipment available, if their workload is manageable and if they are satisfied with their job and their income.^16^

Lack of medication available at the clinics was cited as a reason for patients being unwilling to attend their nearest clinic. This leads to a disruption in the continuity of care and may lead to poor adherence and patient dissatisfaction, as have been reported in previous studies.^13^ Continuity of care was associated with a reduction in resource utilisation and costs.^13^ The chronic care model, which includes healthcare professionals such as pharmacists, has the potential to improve care and reduce costs.^17^

Our study showed that patients – mainly from Soshanguve – who had visited their nearest clinic previously, had access to the same medical services and medication, but still chose KPC over their closest clinic. It seems that these respondents chose KPC for reasons other than financial, transport difficulties or medication availability. It seems likely that patients prefer to use this clinic since it is close to their work or (as many refer family and friends to KPC) that they are happy with the staff's attitude toward them. This was in contrast with other studies in Britain and the USA where patients left their practice because of financial issues.^18^

More women than men attended the KPC clinic. The reason for this was most likely that the women attended in order to use the family-planning services. The majority were aged between 26 and 45 years. Another study, conducted in Liverpool, showed the same results – female patients made up 55.6% of the patients who visited a general practice.^19^ The reason given for this was that the 1966 sample census statistics showed that 52% of the Liverpool population was female.

## Limitations

Some of the clients were afraid to participate as they thought that they might be compromised in their treatment by participating in the study. This fear was minimised by ensuring them of their anonymity and the confidentiality of the data, as well as by explaining that their participation would have no influence on their care at the clinic. All patients at KPC speak and understand either English or Setswana so language did not result in any selection bias.

## Implications of the results

Long queues and long waiting times are a problem in most public-sector primary-care services, but it is of particular concern when it leads to clients being dissatisfied and not using their nearest services. The problem of long queues could be reduced if healthcare centres had enough staff to give quality care to patients and if they organised training for the staff about healthcare practice, which would in turn help with reducing the bad attitude of some of the healthcare providers. Another need is the allocation of larger budgets for medication in the clinics, as well as regular and timely ordering in order to ensure sufficient stock. It would also help if the management of the different healthcare services attended training courses about leadership and health management. Patients at KPC can also be redirected by triage to their nearest clinic, unless there is a need for emergency care.

## Conclusion

The majority of patients who had attended their nearest clinic were adamant that they would not return. However, in order to make the district health system work patients need to be seen at their nearest facility, as planning for these facilities is based on the number of people living in the area. Staff attitudes towards patients need to be improved and there is a need to follow up on patients’ complaints in this regard.

Patients were more happy attending KPC because the staff were not rude to them, the waiting times seemed to be shorter than other clinics and it is in a convenient location, being close to where the majority of the participants work. Patients are so satisfied with the services that they refer more of their friends and family members to KPC, which shows the confidence and trust they have in KPC.
